# Skin permeation mechanism and bioavailability enhancement of celecoxib from transdermally applied nanoemulsion

**DOI:** 10.1186/1477-3155-6-8

**Published:** 2008-07-09

**Authors:** Faiyaz Shakeel, Sanjula Baboota, Alka Ahuja, Javed Ali, Sheikh Shafiq

**Affiliations:** 1 Department of Pharmaceutics, Faculty of Pharmacy, Al-Arab Medical Sciences University, Benghazi-5341, Libya; 2 Department of Pharmaceutics, Faculty of Pharmacy, Jamia Hamdard, Hamdard Nagar, New Delhi-110062, India; 3 New Drug Delivery System (NDDS), Zydus Cadila Research Centre, Ahemdabad, India

## Abstract

**Background:**

Celecoxib, a selective cyclo-oxygenase-2 inhibitor has been recommended orally for the treatment of arthritis and osteoarthritis. Long term oral administration of celecoxib produces serious gastrointestinal side effects. It is a highly lipophilic, poorly soluble drug with oral bioavailability of around 40% (Capsule). Therefore the aim of the present investigation was to assess the skin permeation mechanism and bioavailability of celecoxib by transdermally applied nanoemulsion formulation. Optimized oil-in-water nanoemulsion of celecoxib was prepared by the aqueous phase titration method. Skin permeation mechanism of celecoxib from nanoemulsion was evaluated by FTIR spectral analysis, DSC thermogram, activation energy measurement and histopathological examination. The optimized nanoemulsion was subjected to pharmacokinetic (bioavailability) studies on Wistar male rats.

**Results:**

FTIR spectra and DSC thermogram of skin treated with nanoemulsion indicated that permeation occurred due to the disruption of lipid bilayers by nanoemulsion. The significant decrease in activation energy (2.373 kcal/mol) for celecoxib permeation across rat skin indicated that the stratum corneum lipid bilayers were significantly disrupted (p < 0.05). Photomicrograph of skin sample showed the disruption of lipid bilayers as distinct voids and empty spaces were visible in the epidermal region. The absorption of celecoxib through transdermally applied nanoemulsion and nanoemulsion gel resulted in 3.30 and 2.97 fold increase in bioavailability as compared to oral capsule formulation.

**Conclusion:**

Results of skin permeation mechanism and pharmacokinetic studies indicated that the nanoemulsions can be successfully used as potential vehicles for enhancement of skin permeation and bioavailability of poorly soluble drugs.

## Background

By many estimates up to 90% of new chemical entities (NCEs) discovered by the pharmaceutical industry today and many existing drugs are poorly soluble or lipophilic compounds [1]. The solubility issues obscuring the delivery of these new drugs also affect the delivery of many existing drugs (about 40%). Relative to compounds with high solubility, poor drug solubility often manifests itself in a host of *in vivo *consequences like decreased bioavailability, increased chance of food effect, more frequent incomplete release from the dosage form and higher intersubject variability. Poorly soluble compounds also present many *in vitro *formulation development hindrances, such as severely limited choices of delivery technologies and increasingly complex dissolution testing with limited or poor correlation to the *in vivo *absorption. However, important advances have been made in improving the bioavailability of poorly soluble compounds, so that promising drug candidates need no longer be neglected or have their development hindered by sub optimal formulation. In addition to more conventional techniques, such as micronization, salt formation, complexation etc, novel solubility/bioavailability enhancement techniques have been developed. The recent trend for the enhancement of solubility/bioavailability is lipid based system such as microemulsions, nanoemulsions, solid dispersions, solid lipid nanoparticles and liposomes etc. This is also the most advanced approach commercially, as formulation scientists increasingly turn to a range of nanotechnology-based solutions to improve drug solubility and bioavailability.

Nanoemulsions have been reported to make the plasma concentration profiles and bioavailability of poorly soluble drugs more reproducible [1-5]. Nanoemulsions have also been reported as one of the most promising techniques for enhancement of transdermal permeation and bioavailability of poorly soluble drugs [6-12]. Nanoemulsions are thermodynamically stable transparent (translucent) dispersions of oil and water stabilized by an interfacial film of surfactant and cosurfactant molecules having a droplet size of less than 100 nm [10,11,13]. Many formulation scientists have investigated skin permeation mechanism of many drugs using chemical enhancers [14-21] and microemulsion technique [22,23]. Best of our knowledge, skin permeation mechanism of celecoxib has not been reported using microemulsion or nanoemulsion technique although these techniques have been known to enhance skin permeation of drugs effectively [6-9]. Celecoxib (CXB), a selective cyclo-oxygenase-2 (COX-2) inhibitor has been recommended orally for the treatment of arthritis and osteoarthritis [24]. Long term oral administration of CXB produces serious gastrointestinal side effects [24]. It is a highly lipophilic, poorly soluble drug with oral bioavailability of around 40% (Capsule). Therefore the aim of the present investigation was to evaluate the mechanism of skin permeation and bioavailability of CXB using nanoemulsion technique.

## Materials and methods

### Materials

Celecoxib was a kind gift sample from Ranbaxy Research Labs (India). Propylene glycol mono caprylic ester (Sefsol 218) was a kind gift from Nikko Chemicals (Japan). Diethylene glycol monoethyl ether (Transcutol-P) was gift sample from Gattefosse (France). Glycerol triacetate (Triacetin) and acetonitrile (HPLC grade) were purchased from E-Merck (India). Cremophor-EL was purchased from Sigma Aldrich (USA). Deionized water for HPLC analysis was prepared by a Milli-Q-purification system. All other chemicals used in the study were of analytical reagent grade.

### Preparation of nanoemulsion

Various nanoemulsions were prepared by aqueous phase titration method (spontaneous emulsification method). Optimized nanoemulsion formulation (C2) of CXB was prepared by dissolving 2% w/w of CXB in 15% w/w combination of Sefsol-218 and Triacetin (1:1). Then 35% w/w mixture of Cremophor-EL and Transcutol-P (1:1) were added slowly in oil phase. Then 50% w/w of distilled water was added to get the final preparation.

### Preparation of nanoemulsion gel

Nanoemulsions gel (NGC2) was prepared by dispersing 1% w/w of Carbopol-940 in sufficient quantity of distilled water. This dispersion was kept in dark for 24 h for complete swelling of Carbopol-940. 2% w/w of CXB was dissolved in 15% w/w mixture of Sefsol-218 and Triacetin (1:1). CXB solution was added slowly to Carbopol-940 dispersion. 0.5% w/w of triethanolamine (TEA) was added in this mixture to neutralize Carbopol-940. Then 35% w/w mixture of Cremophor-EL and Transcutol-P (1:1) were added slowly. Then remaining quantity of distilled water was added to get the final preparation 100% w/w.

The composition of nanoemulsion and nanoemulsion gel are given in Table 1.

**Table 1:** Compositions of nanoemulsion (C2) and nanoemulsion gel (NGC2)

**Ingredients**	**C2**	**NGC2**
CXB (% w/w)	2.0	2.0
Carbopol-940 (% w/w)	-	1.0
Sefsol 218 (%w/w)	7.5	7.5
Triacetin (%w/w)	7.5	7.5
Cremophor-EL	17.5	17.5
Transcutol-P (% w/w)	17.5	17.5
Triethanolamine (% w/w)	-	0.5
Distilled water to (% w/w)	100.0	100.0

### Droplet size analysis

Droplet size distribution of optimized nanoemulsion was determined by photon correlation spectroscopy, using a Zetasizer 1000 HS (Malvern Instruments, UK). Light scattering was monitored at 25°C at a scattering angle of 90°. A solid state laser diode was used as light source. The sample of optimized nanoemulsion was suitably diluted with distilled water and filtered through 0.22 μm membrane filter to eliminate mutiscattering phenomena. The diluted sample was then placed in quartz couvette and subjected to droplet size analysis.

### Preparation of full thickness rat skin

Approval to carry out these studies was obtained from the Animal Ethics Committee of Jamia Hamdard, New Delhi, India. Male Wistar rats were sacrificed with prolonged ether anaesthesia and the abdominal skin of each rat was excised. Hairs on the skin of animal were removed with electrical clipper, subcutaneous tissues were surgically removed and dermis side was wiped with isopropyl alcohol to remove residual adhering fat. The skin was washed with distilled water, wrapped in aluminium foil and stored in a deep freezer at -20°C till further use.

### Preparation of epidermis and stratum corneum

The skin was treated with 1 M sodium bromide solution in distilled water for 4 h [25]. The epidermis from full thickness skin was separated using cotton swab moistened with water. Epidermal sheet was cleaned by washing with distilled water and dried under vacuum and examined for cuts or holes if any. Stratum corneum (SC) samples were prepared by floating freshly prepared epidermis membrane on 0.1% trypsin solution for 12 h. Then SC sheets were cleaned by washing with distilled water.

### FTIR spectral analysis of nanoemulsion treated and untreated rat skin

SC was cut into small circular discs. 0.9% w/v solution of sodium chloride was prepared and 0.01% w/v sodium azide was added as antibacterial and antimycotic agent. 35 ml of 0.9% w/v of sodium chloride solution was placed in different conical flasks and SC of approximate 1.5 cm diameter was floated over it for 3 days. After 3 days of hydration, these discs were thoroughly blotted over filter paper and fourier transform infra-red (FTIR) spectra of each SC disc was recorded before nanoemulsion treatment (control) in frequency range of 400 to 4000 cm^-1 ^(Perkin Elmer, Germany). After taking FTIR spectra, the same discs were dipped into CXB nanoemulsion formulation present in 35 ml of methanolic phosphate buffer saline (PBS) pH 7.4 (30:70). This was kept for a period of 24 h (equivalent to the permeation studies) at 37 ± 2°C. Each SC disc after treatment was washed, blotted dry, and then air dried for 2 h. Samples were kept under vacuum in desiccators for 15 min to remove any traces of formulation completely. FTIR spectra of treated SC discs were recorded again. Each sample served as its own control.

### DSC studies of nanoemulsion treated and untreated rat skin

Approximately 15 mg of freshly prepared SC was taken and hydrated over saturated potassium sulphate solution for 3 days. Then the SC was blotted to get hydration between 20 to 25%. Hydrated SC sample was dipped into nanoemulsion formulation present in 35 ml of methanolic PBS pH 7.4 (30:70). This was kept for 24 h (equivalent to the permeation studies) at 37 ± 2°C. After treatment, SC was removed and blotted to attain hydration of 20–25%, cut (5 mg), sealed in aluminum hermatic pans and equilibrated for 1 h before the differential scanning calorimeter (DSC) run. Then, the SC samples were scanned on a DSC6 Differential Scanning Calorimeter (Perkin Elmer, Germany). Scanning was done at the rate of 5°C/min over the temperature range of 30 to 200°C [25,26].

### Determination of activation energy

*In vitro *skin permeation study of CXB across rat skin was carried out at 27, 37, and 47°C in the methanolic PBS pH 7.4 (30:70). These studies were performed on a modified Keshary-Chien diffusion cell with an effective diffusional area of 4.76 cm^2 ^and 35 ml of receiver chamber capacity. In the donor compartment, 1 ml of nanoemulsion formulation was taken (containing 20 mg of CXB). Receiver compartment was composed of the vehicle only (methanolic PBS pH 7.4). Permeability coefficients were calculated at each temperature and activation energy of CXB was then calculated from Arrhenius relationship given as follows [20,27].

P = P_o _e^-Ea/RT ^or

log P = Ea/2.303 RT + log P_o_

Where, Ea is the activation energy, R is gas constant (1.987 kcal/mol), T is absolute temperature in K, P is the permeability cofficient, and Po is the Arrhenius factor.

### Histopathological examination of skin specimens

Abdominal skins of Wistar rats were treated with optimized CXB nanoemulsion (C2) in methanolic PBS pH 7.4. After 24 h, rats were sacrificed and the skin samples were taken from treated and untreated (control) area. Each specimen was stored in 10% formalin solution in methanolic PBS pH 7.4. The specimens were cut into section vertically. Each section was dehydrated using ethanol, embedded in paraffin for fixing and stained with hematoxylin and eosin. These samples were then observed under light microscope (Motic, Japan) and compared with control sample. In each skin sample, three different sites (epidermis, dermis and subcutaneous fat layer) were scanned and evaluated for mechanism of skin permeation enhancement. These slides were interpreted by Dr. Ashok Mukherjee, Professor, Department of Pathology, All India Institute of Medical Sciences (AIIMS), New Delhi, India.

### Pharmacokinetic studies

Approval to carry out pharmacokinetic studies was obtained from the Animal Ethics Committee of Jamia Hamdard, New Delhi, India. Guidelines of ethics committee were followed for the studies. Pharmacokinetic studies were performed on optimized nanoemulsion (C2), nanoemulsion gel (NGC2) and marketed capsule. The male Wistar rats were kept under standard laboratory conditions (temperature 25 ± 2°C and relative humidity of 55 ± 5%). The rats were kept in polypropylene cages (six per cage) with free access to standard laboratory diet (Lipton feed, Mumbai, India) and water *ad libitum*. About 10 cm^2 ^of skin was shaved on the abdominal side of rats in each group except group treated with marketed capsule. They were fasted for the period of 24 h for observations on any unwanted effects of shaving. The dose for the rats was calculated based on the weight of the rats according to the surface area ratio [28]. The rats were divided into 3 groups (n = 6). Group I received C2 transdermally, group II received NGC2 transdermally and group III received marketed capsule orally. The dose of CXB in all groups was 1.78 mg/kg of body weight. The rats were anaesthetized using ether and blood samples (0.5 ml) were withdrawn from the tail vein of rat at 0 (pre-dose), 1, 2, 3, 6, 12, 24, 36, and 48 h in microcentrifuge tubes in which 8 mg of EDTA was added as an anticoagulant. The blood collected was mixed with the EDTA properly and centrifuged at 5000 rpm for 20 min. The plasma was separated and stored at -21°C until drug analysis was carried out using HPLC.

Plasma samples were prepared by adding 500 μl of plasma, 50 μl standard solution of CXB, 50 μl of internal standard solution (ibuprofen), 50 μl of phosphate buffer (pH 5; 0.5 M) and 4 ml of chloroform in small glass tubes. The tubes were vortex for 1 min and centrifuged for 20 min at 5000 rpm. Upper layer was discarded and the chloroform layer was transferred to a clean test tube and evaporated to dryness at 50°C under the stream of nitrogen. The residue was reconstituted in 100 μl of mobile phase, mixed well and 20 μl of the final clear solution was injected into the HPLC system.

CXB in plasma was quantified by the reported HPLC method with slight modifications [29]. The method was validated in our laboratory. A Shimadzu model HPLC equipped with quaternary LC-10A VP pumps, variable wavelength programmable UV/VIS detector SPD-10AVP column oven (Shimadzu), SCL 10AVP system controller (Shimadzu), Rheodyne injector fitted with a 20 μl loop and Class-VP 5.032 software was used. Analysis was performed on a C_18 _column (25 cm × 4.6 mm ID SUPELCO 516 C_18 _DB 5 μm RP-HPLC). The mobile phase consisted of acetonitrile:water (40:60). The mobile phase was delivered at the flow rate of 0.9 ml/min. Detection was performed at 260 nm. Injection volume was 20 μl. The concentration of unknown plasma samples was calculated from the calibration curve plotted between peak area ratios of CXB to IS against corresponding CXB concentrations.

### Pharmacokinetic and statistical analysis

The plasma concentration of CXB at different time intervals was subjected to pharmacokinetic (PK) analysis to calculate various parameters like maximum plasma concentration (C_max_), time to reach maximum concentration (T_max_), and area under the plasma concentration-time curve (AUC_0→t _and AUC_0→ω_). The values of C_max _and T_max _were read directly from the arithmetic plot of time and plasma concentration of CXB. The AUC was calculated by using the trapezoidal method. The relative bioavailability of the CXB after the transdermal administration versus the oral administration was calculated as follows:

$$\text{F }\%=\frac{\text{AUC sample}}{\text{AUC oral}}\cdot \frac{\text{Dose oral}}{\text{Dose sample}}\times 100$$

The PK data between different formulations was compared for statistical significance by one-way analysis of variance (ANOVA) followed by Tukey-Kramer multiple comparisons test using GraphPad Instat software (GraphPad Software Inc., CA, USA).

## Results and discussion

### Droplet size analysis

The mean droplet size of optimized nanoemulsion (C2) was found to be 16.41 ± 1.72 nm. All the droplets were found in the nanometer range which indicated the suitability of formulation for transdermal drug delivery. Polydispersity signifies the uniformity of droplet size within the formulation. The polydispersity value of the formulation C2 was very low (0.105) which indicated uniformity of droplet size within the formulation.

### FTIR spectral analysis of formulation treated and untreated rat skin

**Figure 1:**
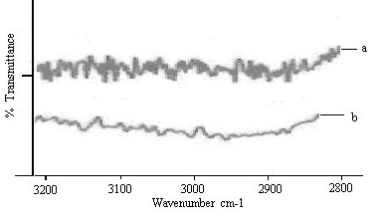
FTIR spectra of rat SC. Change in lipid C-H stretching (2920 cm^-1^) vibrations after 24 hr treatment with (a) control (b) C2.

FTIR spectrum of untreated SC (control) showed various peaks due to molecular vibration of proteins and lipids present in the SC (Figure 1a). The absorption bands in the wave number of 3000 to 2700 cm^-1 ^were seen in untreated SC. These absorption bonds were due to the C-H stretching of the alkyl groups present in both proteins and lipids (Figure 1a). The bands at 2920 cm^-1 ^and 2850 cm^-1 ^were due to the asymmetric -CH_2 _and symmetric -CH_2 _vibrations of long chain hydrocarbons of lipids respectively. The bands at 2955 cm^-1 ^and 2870 cm^-1 ^were due to the asymmetric and symmetric CH_3 _vibrations respectively [30]. These narrow bands were attributed to the long alkyl chains of fatty acids, ceramides and cholesterol which are the major components of the SC lipids.

**Figure 2:**
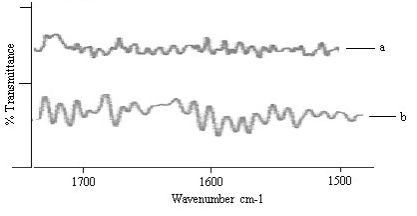
FTIR spectra of rat SC. Change in amide I (1640 cm^-1^) and amide II (1550 cm^-1^) stretching vibrations after 24 h treatment with (a) control (b) C2.

The two strong bands (1650 cm^-1 ^and 1550 cm^-1^)were due to the amide I and amide II stretching vibrations of SC proteins (Figure 2a). The amide I and amide II bands arisen from C = O stretching vibration and C-N bending vibration respectively. The amide I band consisting of components bands, represented various secondary structure of keratin.

There was clear difference in the FTIR spectra of untreated and nanoemulsion treated SC with prominent decrease in asymmetric and symmetric CH- stretching of peak height and area (Figure 1b).

The rate limiting step for transdermal drug delivery is lipophilic part of SC in which lipids (ceramides) are tightly packed as bilayers due to the high degree of hydrogen bonding. The amide I group of ceramide is hydrogen bonded to amide II group of another ceramide and forming a tight network of hydrogen bonding at the head of ceramides. This hydrogen bonding makes stability and strength to lipid bilayers and thus imparts barrier property to SC [31]. When skin was treated with nanoemulsion formulation (C2), ceramides got loosened because of competitive hydrogen bonding leading to breaking of hydrogen bond networks at the head of ceramides due to penetration of nanoemulsion into the lipid bilayers of SC. The tight hydrogen bonding between ceramides caused split in the peak at 1650 cm^-1^(amide I) as shown in the control skin spectrum (Fig 2a). Treatment with nanoemulsion resulted in either double or single peak at 1650 cm^-1^(Figure 2b) which suggested breaking of hydrogen bonds by nanoemulsion.

### DSC studies

**Figure 3:**
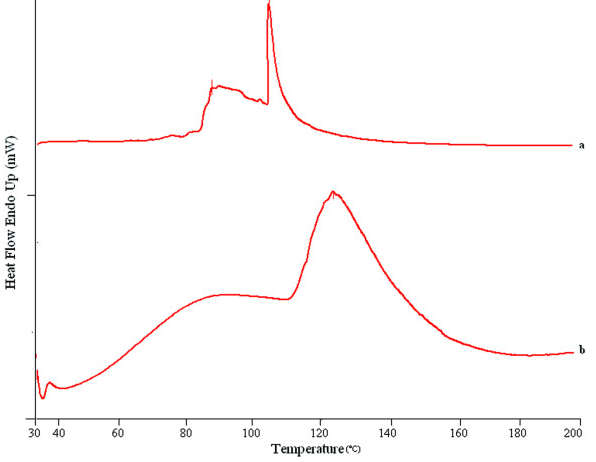
DSC thermogram of control SC and nanoemulsion treated SC for 24 h. (a) control (b) C2.

DSC thermogram of untreated rat epidermis revealed 4 endotherms (Figure 3a). The first 3 endotherms were recorded at 34°C (T_1_), 82°C (C2) and 105°C (T_3_) respectively, whereas fourth endotherm (T_4_) produced a very sharp and prominent peak at 114°C which is attributed to SC proteins. The first endotherm (having the lowest enthalpy) was attributed to sebaceous section [32] and to minor structural rearrangement of lipid bilayer [33]. The second and third endotherm (T2 and T_3_) appeared due to the melting of SC lipids and the fourth endotherm (T_4_) has been assigned to intracellular keratin denaturation [14]. It was observed that both T2 and T_3 _endotherms were completely disappeared or shifted to lower melting points in thermograms of SC treated with nanoemulsion formulation (C2). This indicated that the components (oil, surfactant or cosurfactant) of nanoemulsion enhanced skin permeation of CXB through disruption of lipid bilayers. Nanoemulsion formulation (C2) also decreased the protein endotherm T_4 _to lower melting point, suggesting keratin denaturation and possible intracellular permeation mechanism in addition to the disruption of lipid bilayers (Figure 3b). Thus it was concluded that the intracellular transport is a possible mechanism of permeation enhancement of CXB. Another observation was that T_4_increased up to 122°C in case of nanoemulsion formulation with broadening of the peak. Shift to higher transition temperature (T_m_) and peak broadening has been attributed to dehydration of SC as another mechanism of permeation enhancement in addition to disruption of lipid resulting in higher permeation of CXB [18].

### Determination of activation energy

**Figure 4:**
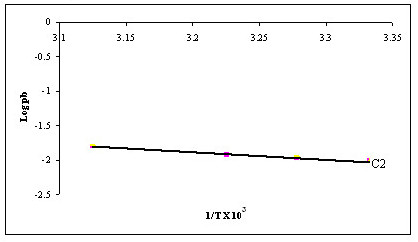
Arrhenius plots of C2 permeation across rat skin.

The activation energy (E_a_) for diffusion of a drug molecule across skin (rat or human) depends on its route of diffusion and physicochemical properties. Nanoemulsions can change this value of E_a _to greater extent by their action on SC lipids. The activation energy for ion transport has been reported as 4.1 and 10.7 kcal/mol across human epidermis [34] and phosphatidylcholine bilayers respectively [35]. The Arrhenius plot between logarithms of permeability coefficient (log P_b_) and reciprocal of absolute temperature (1/T) was found to be linear in the selected temperature range between 27–47°C, indicating no significant structural or phase transition changes within the skin membrane (Figure 4). The value of E_a _for permeation of CXB across rat skin was calculated from the slope of Arrhenius plot. The E_a _of CXB from nanoemulsion formulation C2 was found to be 2.373 kcal/mol. The significant decrease in E_a _for CXB permeation across rat skin indicated that the SC lipid bilayers were significantly disrupted (p < 0.05).

It is also well established that ion transport across skin occurs mainly via aqueous shunt pathways [36]. In the light of these reports it can be anticipated that if a molecule moves via polar pathways across human cadaver epidermis then E_a _value would be akin to that of ion transport across skin. In our study, E_a _of CXB from formulation C2 was 2.373 kcal/mol. Therefore it was concluded that nanoemulsions create pathways in the lipid bilayers of SC resulting in enhanced transdermal permeation of CXB [37].

### Histopathological studies

**Figure 5:**
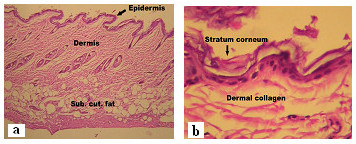
Photomicrographs of skin sample from control group animal showing normal epidermis, dermis and subcutaneous tissues at (a) low power view (HE × 100) (b) high power view (HE × 400).

**Figure 6:**
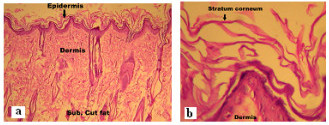
Photomicrographs of skin sample from nanoemulsion treated animal at (a) low power view (HE × 100) (b) high power view (HE × 400).

The photomicrographs of control (untreated skin) showed normal skin with well defined epidermal and dermal layers. Keratin layer was well formed and lied just adjacent to the topmost layer of the epidermis. Dermis was devoid of any inflammatory cells. Skin appendages were within normal limits (Figure 5a&b). When the skin was treated with nanoemulsion formulation (C2) for 24 h, significant changes were observed in the skin morphology. Low power photomicrograph of skin sample showed epidermis with a prominent keratin layer, a normal dermis and subcutaneous tissues. High power photomicrograph of skin sample showed a thickened and reduplicated stratum corneum with up to 8 distinct layers. The epidermis showed increase in its cellular layers to 4–6 cells. Dermis does not show any edema or inflammatory cell infiltration. The disruption of lipid bilayers was clearly evident as distinct voids and empty spaces were visible in the epidermal region (Figure 6a&b). These observations support the *in vitro *skin permeation data of CXB (unpublished data).

There were no apparent signs of skin irritation (erythma and edema etc.) observed on visual examination of skin specimens treated with nanoemulsion formulation.

### Pharmacokinetic studies

**Figure 7:**
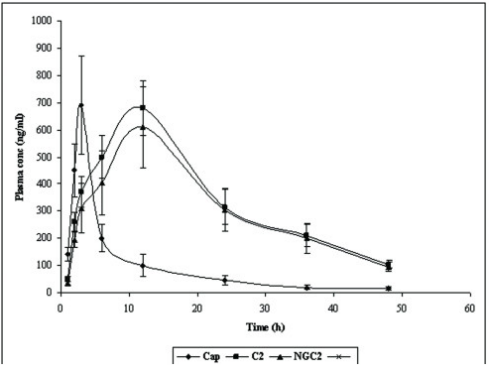
Plasma concentration (Mean ± SD) time profile curve of CXB from C2, NGC2 and capsule (n = 6).

Plasma concentration of CXB from formulations C2, NGC2 and capsule at different time intervals was determined by reported HPLC method. The graph between plasma concentration and time was plotted for each formulation (Fig 7). It was seen from Figure 7 that the plasma concentration profile of CXB for C2 and NGC2 showed greater improvement of drug absorption than the oral capsule formulation. Peak (maximum) plasma concentration (C_max_) of CXB in C2, NGC2 and capsule was 680 ± 100, 610 ± 148 and 690 ± 180 ng/ml respectively whereas time (t_max_) to reach C_max _was 12 ± 2.1, 12 ± 2.4 and 3 ± 0.8 h respectively (Table 2 & Figure 7). AUC_0→t _and AUC_0→ω _in formulations C2, NGC2 and capsule were 14435 ± 1741, 13005 ± 1502 and 4366 ± 1015 ng/ml.h respectively and 19711.3 ± 2012, 17507.3 ± 1654 and 4688.5 ± 1293 ng/ml.h respectively (Table 2). These pharmacokinetic parameters obtained with formulations C2 and NGC2 were significantly different from those obtained with oral capsule formulation (p < 0.05). The significant AUC values observed with C2 and NGC2 also indicated increased bioavailability of the CXB from C2 and NGC2 in comparison with oral capsule formulation (p < 0.05). The formulations C2 and NGC2 were found to enhance the bioavailability of CXB by 3.30 and 2.97 folds (percent relative bioavailability 330 and 297) with reference to the oral capsule (Table 2). This increased bioavailability from transdermal formulations (C2 and NGC2) may be due to the enhanced skin permeation and avoidance of hepatic first pass metabolism.

**Table 2:** Pharmacokinetic parameters (Mean ± SD, n = 6) of CXB from C2, NGC2 and capsule

**Formulation**	**t_max_^a ^± SD** ** (h)**	**C_max_^b ^± SD** ** (ng/ml)**	**AUC_0→t_^c ^± SD ** **(ng/ml.h)**	**AUC_0→α_^d ^± SD ** **(ng/ml.h)**
C2	12 ± 1.8	680 ± 100	14435 ± 1741	19711.3 ± 2012
NGC2	12 ± 2.0	610 ± 148	13005 ± 1502	17507.3 ± 1654
Capsule	3 ± 0.8	690 ± 180	4366 ± 1015	4688.5 ± 1293

^a ^time of peak concentration; ^b ^peak of maximum concentration; ^c ^area under the concentration time profile curve until last observation; ^d ^area under curve extrapolated to infinity

## Conclusion

FTIR spectra and DSC thermogram of skin treated with nanoemulsion indicated that permeation occurred due to the extraction of SC lipids by nanoemulsion. The significant decrease in activation energy for CXB permeation across rat skin indicates that the SC lipid bilayers were significantly disrupted (p < 0.05). Photomicrograph of skin sample showed the disruption and extraction of lipid bilayers as distinct voids and empty spaces were visible in the epidermal region. There were no apparent signs of skin irritation observed on visual examination of skin specimens treated with nanoemulsion formulation. The pharmacokinetic studies revealed significantly greater extent of absorption than the oral capsule formulation (p < 0.05). The absorption of CXB from C2 and NGC2 resulted in 3.30 and 2.97 fold increases in bioavailability as compared to the oral capsule formulation. Results of these studies indicate that nanoemulsions can be successfully used for enhancement of skin permeation as well as bioavailability of poorly soluble drugs.

## Abbreviations

FTIR: Fourier transforms infra-red; DSC: Differential scanning calorimetry; CXB: Celecoxib; SC: Stratum corneum; C_max: _Peak or maximum plasma concentration; T_max:_ Time to reach peak plasma concentration; AUC: Area under plasma concentration time profile curve; NCEs: New chemical entities; COX-2: Cyclo-oxygenase-2; HPLC: High performance liquid chromatography; C2: Optimized nanoemulsion; NGC2: Nanoemulsion gel; PBS: Phosphate buffer saline; AIIMS: All india institute of medical sciences; EDTA: Ethylene diamine tetra-acectic acid; rpm: Revolution per minute; min: Minutes; IS: Internal standard; RP-HPLC: Reverse phase high performance liquid chromatography; PK: Pharmacokinetic; AUC_0→t:_ Area under curve from time o to t; AUC_0→ω: _Area under curve from time o to infinitive; % F: Percent relative bioavailability; ANOVA: Analysis of variance.

## Competing interests

The authors declare that they have no competing interests.

## Authors' contributions

FS performed pharmacokinetic studies. SB and AA prepared skin for Histopathological examination and activation energy measurement. JA took FTIR spectra and DSC thermogram. SS validated HPLC method for analysis of drug in plasma samples. SB, AA and JA guided the studies. Finally manuscript has been checked and approved by all the authors.
